# Longitudinal Intraindividual Cognitive Variability Is Associated With Reduction in Regional Cerebral Blood Flow Among Alzheimer’s Disease Biomarker-Positive Older Adults

**DOI:** 10.3389/fnagi.2022.859873

**Published:** 2022-07-06

**Authors:** Sophia L. Holmqvist, Kelsey R. Thomas, Einat K. Brenner, Emily C. Edmonds, Amanda Calcetas, Lauren Edwards, Maria Bordyug, Katherine J. Bangen

**Affiliations:** ^1^Research Service, VA San Diego Healthcare System, San Diego, CA, United States; ^2^Department of Psychiatry, University of California, San Diego, La Jolla, CA, United States; ^3^San Diego State University/University of California San Diego Joint Doctoral Program in Clinical Psychology, San Diego, CA, United States

**Keywords:** neuropsychology, intra-individual variability, aging, cerebral blood flow (CBF), magnetic resonance imaging (MRI), Alzheimer’s disease, phosphorylated tau (p-tau), amyloid beta

## Abstract

Intraindividual variability (IIV) across neuropsychological measures within a single testing session is a promising marker predictive of cognitive decline and development of Alzheimer’s disease (AD). We have previously shown that greater IIV is cross-sectionally associated with reduced cerebral blood flow (CBF), but not with cortical thickness or brain volume, in older adults without dementia who were amyloid beta (Aβ) positive. However, there is little known about the association between change in IIV and CBF over time. Therefore, we examined 12-month longitudinal change in IIV and interactions of IIV and AD biomarker status on changes in regional CBF. Fifty-three non-demented Alzheimer’s Disease Neuroimaging Initiative (ADNI) participants underwent lumbar puncture to obtain cerebrospinal fluid (CSF) at baseline and neuropsychological testing and magnetic resonance imaging (MRI) exams at baseline and 12-month follow-up evaluation. IIV was calculated as the intraindividual standard deviation across 6 demographically-corrected neuropsychological measures. Pulsed arterial spin labeling (ASL) MRI was acquired to quantify CBF and FreeSurfer-derived *a priori* CBF regions of interest (ROIs) were examined. AD biomarker positivity was determined using a published CSF p-tau/Aβ ratio cut-score. Change scores were calculated for IIV, CBF, and mean neuropsychological performance from baseline to 12 months. Hierarchical linear regression models showed that after adjusting for age and gender, there was a significant interaction between IIV change and biomarker-positivity (p-tau/Aβ+) for change in entorhinal and hippocampal CBF but not for the other ROIs. Specifically, increases in IIV were associated with reductions in entorhinal and hippocampal CBF among individuals who were biomarker-positive (*n* = 21). In contrast, there were no significant associations between change in IIV and CBF among those who were biomarker-negative (*n* = 32). Findings remained similar when analyses were performed adjusting for change in mean level of neuropsychological performance. Changes in IIV may be sensitive to changes in regional hypoperfusion in AD-vulnerable regions among AD biomarker-positive individuals, above and beyond demographics and mean neuropsychological performance. These findings provide further evidence supporting IIV as a potential marker of cerebrovascular brain changes in individuals at risk for dementia.

## Introduction

There is a strong need to identify those who are at risk for dementia prior to the onset of significant cognitive and functional decline. Previous research in preclinical AD suggests there may be temporal overlap in the development of neuropathologic and cognitive changes (Braak et al., 2013) and subtle cognitive difficulties may be as reliable as other AD biomarkers, including Aβ, in predicting progression to MCI and AD (Edmonds et al., 2015a). A common approach to detecting cognitive impairment in suspected early AD has been to examine mean episodic memory performance (Derby et al., 2013), however, there are limitations of this approach. Subtle changes in cognitive performance, such as reduced efficiency or more errors, may be more sensitive than overall mean score very early in the disease process and may be particularly useful among individuals who have high premorbid cognitive abilities and for who subtle decline may go undetected if determined based on overall mean scores (Kälin et al., 2014; Thomas et al., 2018). There has been evidence showing that intraindividual cognitive variability (IIV), or variability in neuropsychological performance across measures in a single testing session (Stuss et al., 2003; Koscik et al., 2016; Malek-Ahmadi et al., 2017; Gleason et al., 2018; Bangen et al., 2019), may be sensitive to early subtle changes in cognition before conventional cognitive criteria can be met (Stuss et al., 2003).

While some variability in domains of cognitive performance is normal, greater variability has been associated with decreased neuronal integrity (Lovden et al., 2013), subcortical ischemic vascular cognitive impairment (Richards et al., 2019), greater dementia incidence at follow-up (Watermeyer et al., 2020), and having an AD classification (Halliday et al., 2018). However, *in vivo* brain changes underlying IIV have received relatively limited study. The few studies linking IIV to brain changes have shown that greater IIV relates to faster rates of cerebral atrophy in entorhinal cortex and hippocampus at follow-up (Bangen et al., 2019) and more severe neurofibrillary tangle pathology and neuritic plaques (Malek-Ahmadi et al., 2017). In contrast, another recent study found that IIV was not associated with cerebrospinal fluid (CSF) based markers of amyloid (Watermeyer et al., 2020). There have been no studies published to date examining baseline IIV associations with early cerebrovascular damage such as reduced cerebral blood flow (CBF) using arterial spin labeling (ASL) magnetic resonance imaging (MRI).

Cerebral blood flow (CBF) is quantified as the rate of delivery of arterial blood to capillary beds. ASL MRI, a non-invasive method for measuring CBF, uses magnetically labeled arterial water as an endogenous tracer (Liu and Brown, 2007). The co-occurrence of both cerebrovascular and AD neuropathologies in clinically diagnosed mild cognitive impairment and AD dementia is very common (Kapasi et al., 2017), and it has been theorized that vascular dysfunction is one of the earliest changes in AD pathogenesis, in which hypoperfusion and blood brain barrier dysfunction pave the way for further vascular damage and subsequent accumulation of amyloid pathology (Zlokovic, 2011; Iturria-Medina et al., 2016; Kisler et al., 2017). Evidence has shown that early changes in CBF predict conversion to dementia (Wierenga et al., 2014; Sanchez et al., 2020) and that these early changes precede brain atrophy and the accumulation of white matter hyperintensities, an MRI marker of small vessel cerebrovascular disease (Bangen et al., 2021).

In this study, we investigated associations between longitudinal change in IIV and CBF as well as interactions of IIV change and AD biomarker status on changes in regional CBF in older adults without dementia. We hypothesized that a greater increase in IIV over 12 months would be associated with lower CBF at 12 months in AD vulnerable brain regions, particularly among individuals who are thought to be on the AD continuum based on positive CSF biomarkers.

## Materials and Methods

### The ADNI Dataset

Data used in the preparation of this article were obtained from the Alzheimer’s Disease Neuroimaging Initiative (ADNI) database^1^. The ADNI was launched in 2003 as a public-private partnership, led by Principal Investigator Michael W. Weiner, MD. The primary goal of ADNI has been to test whether serial magnetic resonance imaging (MRI), positron emission tomography (PET), other biological markers, and clinical and neuropsychological assessment can be combined to measure the progression of mild cognitive impairment (MCI) and early Alzheimer’s disease (AD). For up-to-date information, see www.adni-info.org.

### Participants

Specific enrollment criteria for ADNI have been previously described in detail elsewhere (Petersen et al., 2010). Briefly, participants from ADNI were 55–90 years old, had ≥ 6 years of education or work-history equivalent, were fluent in English or Spanish, had a Geriatric Depression Scale < 6, had a Hachinski Ischemia Scale < 5, had adequate vision and hearing to perform neuropsychological tests, were in generally good health and without significant head trauma or neurologic disease, were stable on permitted medications, and had a reliable study partner. The current study included 53 participants from ADNI GO/ADNI 2 cohorts when ASL MRI was collected. Participants were included if they had ASL and neuropsychological data collected at their baseline visit and 12 months follow-up, did not have dementia at their baseline study visit, and underwent lumbar puncture for cerebrospinal fluid collection at baseline. See Supplementary Material for study sample flow chart.

Participants were characterized by ADNI as having normal cognition, significant memory complaint (SMC), early mild cognitive impairment (EMCI), or late MCI (LMCI) (Petersen et al., 2010). ADNI’s SMC diagnosis is based on having a significant memory concern but an unimpaired delayed recall score of Story A from Wechsler Memory Scale-Revised (WMS-R) Logical Memory II. ADNI’s MCI diagnoses were based on the following (1) subjective memory concern reported by the participant, study partner, or clinician; (2) abnormal memory function documented by scoring within education-adjusted ranges on delayed free recall of Story A from the WMS-R Logical Memory II subtest; (3) Mini-Mental State Examination (MMSE) score between 24 and 30; (4) global Clinical Dementia Rating (CDR) score of 0.5, with a Memory Box score of at least 0.5; and (5) general cognition and functional performance sufficiently preserved such that a diagnosis of dementia could not be made. All MCI participants who met these criteria were further classified as “early MCI” or “late MCI” by ADNI based on education-adjusted cut-offs on WMS-R Logical Memory II Story A. Finally, participants were classified as cognitively normal based on (1) no memory complaints, beyond what would be expected for age; (2) normal memory function documented by scoring above education-adjusted cutoffs on delayed free recall of Story A from WMS-R Logical Memory II (score of ≥ 9 for 16 or more years of education, score of ≥ 5 for 8–15 years of education, or score of ≥ 3 for 0–7 years of education); (3) MMSE score between 24 and 30; (4) global CDR of 0; and (5) no significant impairment in cognitive functioning or activities of daily living (Petersen et al., 2010).

This study was approved by the Institutional Review Board at the ADNI study sites. Treatment of human participants during this study was in full accordance with ethical standards set forth by the Helsinki Declaration. Informed written consent was obtained from all participants at each study site.

### Intra-Individual Cognitive Variability

The IIV index is the variability across cognitive measures at a single time point. We calculated the index of dispersion, or IIV, using procedures previously described (Bangen et al., 2019). The IIV index consisted of standard summary measures from tests designed to assess multiple different cognitive abilities. Six neuropsychological measures were selected given their routine use in assessing early cognitive changes in AD, administration across all ADNI waves, and sampling of three different domains of cognition (i.e., language, processing speed/executive function, and episodic memory). These six measures were: (1) Animal Fluency, total score; (2) 30-item Boston Naming Test (BNT), total score; (3) Trail Making Test (TMT), Part A; time to completion, (4) TMT, Part B; time to completion, (5) Rey Auditory Verbal Learning Test (AVLT) 30-min delayed free recall; number of words recalled, and (6) AVLT recognition; number of words correctly recognized.

Individual raw scores for each measure were converted into age-, gender-, and education-adjusted z-scores with a mean of 0 and a standard deviation of 1 using regression coefficients derived from robust cognitively normal participants (*n* = 385) who had at least 1 year of follow up and remained cognitively normal throughout their participation in the ADNI study (Edmonds et al., 2015b). The TMT scores were multiplied by −1 so that higher z-scores represent better performance for all scores. The intraindividual standard deviation across these 6 z-scores was used to quantify IIV. A higher IIV index indicates greater variability across neuropsychological measures in a single session whereas a low IIV index indicates more consistency across measures. Mean level of cognitive performance was derived as the average of the 6 z-scores that were included in the IIV index. Change in IIV and mean neuropsychological performance were calculated as the difference between IIV and mean neuropsychological performance values from study baseline to 12 months follow-up.

### T1-Weighted Anatomical and Arterial Spin Labeling MRI Data Acquisition and Processing

Detailed information describing the imaging data acquisition and processing is available online at www.loni.usc.edu. Structural MRI and ASL scans were collected during the same session on a 3.0 Tesla Siemens scanner (Jack et al., 2015). FreeSurfer Version 5.1 was used to motion correct, skull strip, segment, and parcellate brain regions from structural scans^2^ (Fischl et al., 2002, 2004).

Pulsed ASL scans were collected using QUIPS II with thin-slice TI1 periodic saturation with echo-planar imaging (Luh et al., 1999). The scan parameters are as follows: inversion time of arterial spins (TI1) = 700 ms, total transit time of spins (TI2) = 1,900 ms, tag thickness = 100 mm, tag to proximal slice gap = 25.4 mm, repetition time = 3,400 ms, echo time = 12 ms, field of view = 256 mm, matrix = 64 × 64, 24 4-mm thick axial slices [52 tag + control image pairs], time lag between slices = 22.5 ms.

ASL data processing involved automated motion correction, aligning each ASL frame to the first frame using a rigid body transformation, and least squares fitting using SPM 8^3^ as described previously (Sanchez et al., 2020; Thomas et al., 2020; Bangen et al., 2021). The difference of the mean-tagged and mean-untagged ASL images were calculated for the perfusion-weighted images. The images were intensity scaled to account for signal decay during acquisition and to generate intensities in meaningful physiological units. ASL images were aligned to structural T1 images using FSL after geometric distortion correction. A partial volume correction was performed that assumed that CBF in gray matter is 2.5 times greater than in white matter to mitigate effects of lower perfusion in white matter on CBF estimates. These images were normalized by the reference image (i.e., an estimate of blood water magnetization) to convert the signal into physical units (mL/100 g tissue/min). After, a global pass/fail rating was given based on visual inspection of signal uniformity, geometrical distortions, gray matter contrast, and presence of large artifacts for ADNI quality control purposes. A rating of “unusable” in any of these categories resulted in a global “fail” and that participant was excluded from this study.

To extract regional CBF estimates for each participant, FreeSurfer-derived anatomical regions of interest (ROIs) were applied to CBF maps. Our analyses examined the following six *a priori* ROIs: (1) hippocampus, (2) inferior parietal lobe (IPL), (3) inferior temporal gyrus (ITG), (4) medial orbitofrontal cortex (mOFC), (5) rostral middle frontal gyrus (rMFG), and (6) entorhinal cortex. These regions were selected given prior findings showing these regions are prone to early AD-related change (Dickerson et al., 2011) and consistent with our previous studies examining CBF in ADNI (e.g., Sanchez et al., 2020; Thomas et al., 2020). CBF values were residualized by precentral CBF which was used as the reference region as it is not thought to be impacted in early AD (allowing for adjustment of individual variability in CBF that is likely not due to AD pathologies) as well as to be concordant with previous ADNI ASL studies that used this approach (Mattsson et al., 2014; Yew et al., 2017). Mean CBF corrected for partial volume effects was extracted for each of the ROIs and the reference region for each hemisphere separately. Averaged bilateral CBF estimates for each ROI were used as the dependent variable in analyses to minimize the number of statistical comparisons. Bilateral CBF estimates were derived by averaging the mean CBF of each hemisphere. Change in CBF for each ROI was calculated as the difference between regional CBF values at study baseline and 12 months follow-up.

### Cerebrospinal Fluid Biomarkers

Cerebrospinal fluid biomarkers of amyloid-β (Aβ) and hyperphosphorylated tau (p-tau) were processed with Elecsys^®^ immunoassays. AD biomarker positivity was determined using a previously determined p-tau/Aβ ratio cut-off score (Schindler et al., 2018).

### Statistical Analyses

Analysis of variance (ANOVA) or Chi-squared (χ^2^) tests examined baseline differences in demographic and clinical characteristics between the AD biomarker-positive and biomarker-negative groups. Hierarchical linear regression, adjusting for age and gender, was used to examine the associations between the IIV index, CSF p-tau/Aβ positivity, and change in CBF from baseline to 12 months in the six *a priori* ROIs: hippocampus, IPL, ITG, mOFC, entorhinal, and rMFG. For all models, age and gender were entered on Step 1; p-tau/Aβ status and mean centered change in IIV were entered on step 2; and the interaction of p-tau/Aβ status and mean centered change in IIV was entered on step 3. In secondary models, change in mean level of cognitive performance was added as a covariate to see if results would remain. All analyses were performed using Statistical Package for the Social Sciences (SPSS) version 28 (SPSS IBM, NY, United States). An alpha = 0.05 was set for statistical significance.

Finally, to address potential inflation of type I error resulting from multiple comparisons, we applied the Benjamini–Hochberg procedure (Benjamini and Hochberg, 1995). We assessed results when the false discovery rate (FDR) was controlled at 0.10.

## Results

### Participant Characteristics

Participants’ baseline demographic and clinical data are presented in Table 1. Fifty-three older adults ranging in age from 55 to 85 (mean ± SD = 70.98 ± 6.3) comprised the present sample. There were 27 women (50.8%), the sample was 100% white, and the average number of years of formal education was approximately 17 (SD = 2.53). Compared to p-tau/Aβ– participants, the p-tau/Aβ+ group was significantly older and had poorer cognitive functioning (i.e., lower mean level of cognitive performance and poorer performance on each of the individual cognitive measures). There were no significant group differences in terms of education or gender (*p-values* > 0.05). The p-tau/Aβ+ group showed qualitatively higher IIV at baseline [mean (SD) IIV = 0.93 (0.45) vs. 0.77 (0.27), p = 0.12] and significantly higher IIV at 12-month follow-up [mean (SD) IIV = 0.97 (0.41) vs. 0.73 (0.23), p = 0.01] compared to p-tau/Aβ– individuals.

**TABLE 1:** Baseline demographics for overall sample and by p-tau/Aβ biomarker status.

	Entire sample (*n* = 53)	p-tau/Aβ+ (*n* = 21)	p-tau/Aβ– (*n* = 32)	F or χ^2^	p	Effect size
Age, years	70.46 (7.33)	72.96 (7.56)	68.79 (6.79)	4.43	**0.040**	0.080
Education, years	16.83 (2.53)	16.48 (2.82)	17.06 (2.33)	0.68	0.414	0.013
Gender (% Women)	50.9%	47.6%	53.1%	0.15	0.695	−0.054
p-tau/Aβ+ (%) ^a^	39.6%	–	–	–	–	
Cognitive status (%)				3.31	0.347	0.250
Cognitively normal	32.1%	23.8%	37.5%			
SMC	1.9%	0.0%	3.1%			
Early MCI	35.8%	33.3%	37.5%			
Late MCI	30.2%	42.9%	21.9%			
Mean Cognitive Performance	−0.37 (0.71)	−0.79 (0.87)	−0.09 (0.40)	14.98	**< 0.001**	0.227
IIV	0.83 (0.35)	0.93 (0.45)	0.77 (0.27)	2.56	0.115	0.048
Animal Fluency	−0.41 (0.76)	−0.71 (0.56)	−0.22 (0.82)	5.63	**0.021**	0.099
Boston Naming Test	−0.21 (0.96)	−0.61 (1.24)	0.05 (0.61)	6.74	**0.012**	0.117
Trail Making Test, Part A	−0.05 (1.20)	−0.27 (1.63)	0.09 (0.82)	1.11	0.297	0.021
Trail Making Test, Part B	−0.14 (1.05)	−0.47 (1.38)	0.06 (0.74)	3.22	0.079	0.062
AVLT Delayed Recall	−0.68 (1.08)	−1.35 (0.91)	−0.24 (0.95)	17.71	**< 0.001**	0.258
AVLT Recognition	−0.69 (1.26)	−1.25 (1.39)	−0.33 (1.03)	7.70	**0.008**	0.131

*Results from analysis of variance (ANOVAs) for continuous variables and chi-square tests for dichotomous variables. Data are summarized as mean (standard deviation), unless otherwise indicated. Significant group differences (p < 0.05) appear in bold font. Effect sizes are partial eta-squared for ANOVAs or Phi for chi-square tests. SD, standard deviation; p-tau, phosphorylated tau; Aβ, amyloid beta; SMC, significant memory concern; MCI, mild cognitive impairment; IIV, intraindividual cognitive variability; AVLT, Rey Auditory Verbal Learning Test; ^a^p-tau/amyloid negativity vs. positivity was determined using a published CSF p-tau/Aβ ratio cut-off value (Schindler et al., 2018).*

*^b^Mean cognitive performance is the baseline mean of the six age-, gender-, and education-adjusted neuropsychological z scores included in the IIV index. The six scores were baseline Animal Fluency, total score; 30-item Boston Naming Test (BNT) total score; Trail Making Test (TMT), Part A time to completion; TMT, Part B time to completion; Rey Auditory Verbal Learning Test (AVLT) 30-min delayed free recall number of words recalled; and AVLT recognition number of words correctly recognized.*

### Associations of IIV Change, Alzheimer’s Disease Biomarker Positivity, and Regional Cerebral Blood Flow

**FIGURE 1:**
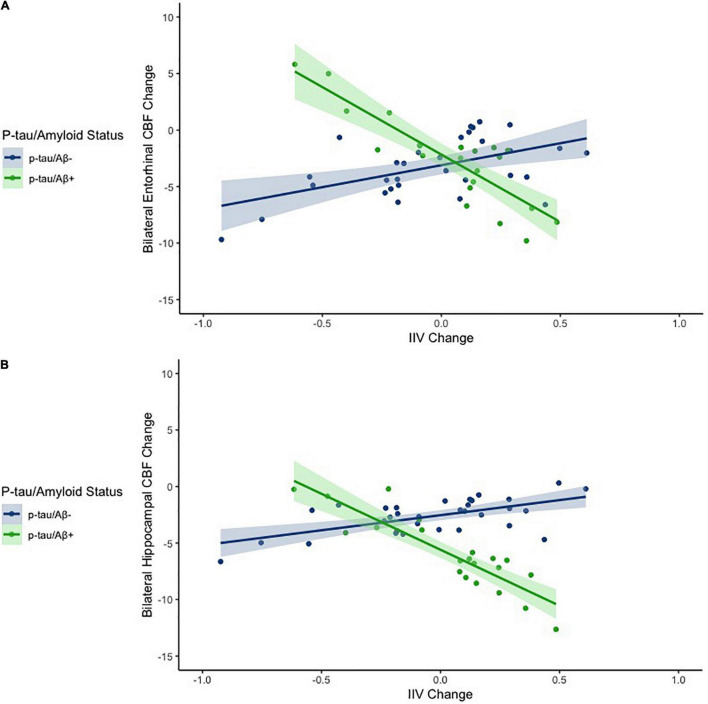
Scatterplots depicting the association between IIV change and CBF change across the entire sample for entorhinal cortex **(A)** and the hippocampus **(B)**. The x axes depict IIV change values. The y axes depict model predicted regional CBF change (residualized by precentral CBF). Models are adjusted for age and sex.

Hierarchical linear regression models showed that after adjusting for age and gender, as well as for the main effects of IIV change and p-tau/Aβ status, there was a significant interaction between IIV change and p-tau/Aβ status for change in entorhinal [β = −0.335, *p* = 0.047; Overall model: R^2^ = 0.175, F (5,47) = 1.995, *p* = 0.097] and hippocampal CBF [β = −0.380, *p* = 0.020; Overall model: R^2^ = 0.256, F (5,47) = 3.241, *p* = 0.014], but not for IPL, ITF, mOFC, or rMFG (*p*-values > 0.05). There were no main effects of p-tau/Aβ status or IIV change across the ROIs (see Table 2 and Figure 1). When FDR was applied, the significant finding for hippocampal CBF was maintained although the finding for entorhinal CBF were not.

**TABLE 2:** Results of hierarchical linear regression models examining interactions of IIV change and AD biomarkers on regional CBF.

	Hippocampal CBF			IPL CBF			ITG CBF			mOFC CBF			rMFG CBF			Entorhinal CBF		
**Block 1**	R^2^ = 0.104 *F* = 2.892 *p* = 0.065			R^2^ = 0.173 *F* = 5.01 ***p* = 0.011**			R^2^ = 0.131 *F* = 3.764 ***p* = 0.030**			R^2^ = 0.111 *F* = 3.128 *p* = 0.052			R^2^ = 0.048 *F* = 1.272 *p* = 0.289			R^2^ = 0.088 *F* = 2.425 *p* = 0.099		

	**B**	**S.E.**	**p**	**B**	**S.E.**	**p**	**B**	**S.E.**	**p**	**B**	**S.E.**	**p**	**B**	**S.E.**	**p**	**B**	**S.E**	**p**

Age	−0.251	0.105	**0.020**	−0.445	0.141	**0.003**	−0.328	0.128	**0.013**	−0.278	0.114	**0.018**	−0.255	0.162	0.122	−0.181	0.143	0.213
Gender	0.590	1.520	0.699	1.119	1.990	0.577	−1.293	1.855	0.489	1.310	1.651	0.431	−0.257	2.356	0.913	−3.457	2.078	0.102
**Block 2**	R^2^ = 0.162 R^2^ change = 0.058 *F* = 2.319 *p* = 0.070			R^2^ = 0.207 R^2^ change = 0.035 *F* = 3.008 ***p* = 0.028**			R^2^ = 0.201 R^2^ change = 0.070 *F* = 3.025 ***p* = 0.026**			R^2^ = 0.131 R^2^ change = 0.019 *F* = 1.802 *p* = 0.144			R^2^ = 0.0.086 R^2^ change = 0.038 *F* = 1.136 *p* = 0.351			R^2^ = 0.102 R^2^ change = 0.013 *F* = 1.359 *p* = 0.262		
Age	−0.222	0.110	**0.049**	−0.428	0.147	**0.006**	−0.305	0.133	**0.027**	−0.269	0.122	**0.033**	−0.204	0.173	0.245	−0.225	0.155	0.153
Gender	0.395	1.505	0.794	1.053	1.993	0.600	−1.519	1.821	0.409	1.211	1.673	0.473	−0.527	2.365	0.825	−3.342	2.114	0.120
p-tau/Aβ	−2.251	1.620	0.171	−1.582	2.124	0.460	−2.528	1.96	0.203	−1.111	1.800	0.540	−3.182	2.546	0.217	1.506	2.275	0.511
IIV Change	−2.187	2.359	0.359	−3.494	3.243	0.287	−3.868	2.854	0.182	−1.857	2.62	0.482	−1.605	3.706	0.667	−2.083	3.311	0.532
**Block 3**	R^2^ = 0.256 R^2^ change = 0.094 *F* = 3.241 ***p* = 0.014**			R^2^ = 0.208 R^2^ change = 0.001 *F* = 2.367 *P* = 0.054			R^2^ = 0.204 R^2^ change = 0.003 *F* = 2.408 *p* = 0.050			R^2^ = 0.144 R^2^ change = 0.014 *F* = 1.586 *p* = 0.183			R^2^ = 0.0.092 R^2^ change = 0.006 *F* = 0.955 *p* = 0.455			R^2^ = 0.175 R^2^ change = 0.073 *F* = 1.995 *p* = 0.097		
Age	−0.195	0.105	0.071	−0.422	0.151	0.008	−0.299	0.135	**0.032**	−0.257	0.123	**0.043**	−0.214	0.175	0.229	−0.192	0.151	0.209
Gender	0.626	1.436	0.665	1.048	2.014	0.605	−1.471	1.842	0.428	1.307	1.680	0.441	−0.613	2.388	0.798	−3.066	2.051	0.142
p-tau/Aβ	−1.834	1.552	0.243	−1.496	2.178	0.496	−2.443	1.99	0.226	−0.937	1.816	0.608	−3.338	2.58	0.202	2.003	2.216	0.371
IIV Change	1.576	2.723	0.565	−2.963	4.007	0.464	−3.098	3.492	0.379	−0.288	3.186	0.928	−3.010	4.527	0.509	2.414	3.889	0.538
IIV Change x p-tau/Aβ	−11.718	4.796	**0.018**	−1.634	7.098	0.819	−2.398	6.151	0.698	−4.887	5.612	0.388	4.374	7.975	0.586	−14.002	6.850	**0.047**

*p-tau, phosphorylated tau; Aβ, amyloid beta; S.E., standard error; IIV, intraindividual cognitive variability; CBF, cerebral blood flow; IPL, inferior parietal lobe; ITG, inferior temporal gyrus; mOFC, medial orbitofrontal cortex; rMFG, rostral middle frontal gyrus. For gender, women are the reference group. Bold values are statistically significant (p < 0.05). IIV was calculated by using the intraindividual standard deviation across 6 z-scores.*

When analyses were stratified by AD biomarker status, increases in IIV were associated with reductions in entorhinal [β = −0.509, *p* = 0.007; Overall model: R^2^ = 0.369, F (3,17) = 3.307, *p* = 0.045] and hippocampal CBF [β = −0.447, *p* = 0.055; Overall model: R^2^ = 0.208, F (3,17) = 1.488, *p* = 0.253] among individuals who were p-tau/Aβ+ (*n* = 21) (see Table 3). In contrast, there were no significant associations between change in IIV and CBF among those who were biomarker-negative (p-tau/Aβ–; *n* = 32) (see Table 4). The significant association between IIV and entorhinal CBF was maintained when the FDR was applied.

**TABLE 3:** Results of hierarchical linear regression models examining associations of IIV change and regional CBF in p-tau/Aβ+ older adults.

	Hippocampal CBF			IPL CBF			ITG CBF			mOFC CBF			rMFG CBF			Entorhinal CBF		
**Block 1**	R^2^ = 0.010 *F* = 0.088 *p* = 0.916			R^2^ = 0.143 *F* = 1.414 *p* = 0.270			R^2^ = 0.223 *F* = 2.584 *p* = 0.103			R^2^ = 0.129 *F* = 1.337 *p* = 0.288			R^2^ = 0.096 *F* = 0.959 *p* = 0.402			R^2^ = 0.020 *F* = 0.179 *p* = 0.837		

	**B**	**S.E**	**p**	**B**	**S.E**	**p**	**B**	**S.E**	**p**	**B**	**S.E.**	**p**	**B**	**S.E.**	**p**	**B**	**S.E**	**p**

Age	−0.020	0.200	0.922	−0.516	0.307	0.111	−0.476	0.232	0.055	−0.321	0.204	0.132	−0.278	0.279	0.332	−0.078	0.182	0.674
Gender	−1.216	2.947	0.685	0.438	4.354	0.921	−3.634	3.421	0.302	1.086	3.009	0.722	−4.137	4.115	0.328	−1.174	2.682	0.667
**Block 2**	R^2^ = 0.208 R^2^ change = 0.198 *F* = 1.488 *p* = 0.253			R^2^ = 0.158 R^2^ change = 0.015 *F* = 0.999 *p* = 0.418			R^2^ = 0.257 R^2^ change = 0.034 *F* = 1.960 *p* = 0.158			R^2^ = 0.177 R^2^ change = 0.047 *F* = 1.217 *p* = 0.334			R^2^ = 0.0.100 R^2^ change = 0.065 *F* = 0.628 *p* = 0.607			R^2^ = 0.369 R^2^ change = 0.349 *F* = 3.307 ***p* = 0.005**		
Age	−0.029	0.184	0.878	−0.499	0.316	0.134	−0.481	0.233	0.055	−0.326	0.204	0.128	−0.276	0.286	0.348	−0.088	0.150	0.564
Gender	−0.719	2.723	0.795	0.472	4.448	0.917	−3.364	3.456	0.344	1.351	3.023	0.661	−4.232	4.243	0.332	−0.570	2.223	0.801
IIV Change	−9.815	4.758	0.055	−4.368	8.137	0.599	−5.325	6.039	0.390	−5.23	5.281	0.336	1.888	7.413	0.802	−11.908	3.885	**0.007**

*p-tau, phosphorylated tau; Aβ, amyloid beta; S.E., standard error; IIV, intraindividual cognitive variability; CBF, cerebral blood flow; IPL, inferior parietal lobe; ITG, inferior temporal gyrus; mOFC, medial orbitofrontal cortex; rMFG, rostral middle frontal gyrus. For gender, women are the reference group. Bold values are statistically significant (p < 0.05). IIV was calculated by using the intraindividual standard deviation across 6 z-scores.*

**TABLE 4:** Results of hierarchical linear regression models examining associations of IIV change and regional CBF in p-tau/Aβ–older adults.

	Hippocampal CBF			IPL CBF			ITG CBF			mOFC CBF			rMFG CBF			Entorhinal CBF		
**Block 1**	R^2^ = 0.283 *F* = 5.715 ***p* = 0.008**			R^2^ = 0.168 *F* = 2.824 *p* = 0.076			R^2^ = 0.032 *F* = 0.475 *p* = 0.627			R^2^ = 0.054 *F* = 0.822 *p* = 0.450			R^2^ = 0.026 *F* = 0.392 *p* = 0.679			R^2^ = 0.141 *F* = 2.379 *p* = 0.111		

	**B**	**S.E.**	**p**	**B**	**S.E.**	**p**	**B**	**S.E.**	**p**	**B**	**S.E.**	**p**	**B**	**S.E.**	**p**	**B**	**S.E**	**P**

Age	−0.384	0.115	**0.002**	−0.331	0.139	**0.025**	−0.123	0.155	0.435	−0.193	0.152	0.216	−0.168	0.215	0.442	−0.282	0.231	0.232
Gender	2.116	1.540	0.180	1.200	1.850	0.522	−0.736	2.079	0.726	1.052	2.039	0.610	1.724	2.875	0.553	−4.478	3.089	0.158
**Block 2**	R^2^ = 0.285 R^2^ change = 0.002 *F* = 3.719 ***p* = 0.023**			R^2^ = 0.193 R^2^ change = 0.026 *F* = 2.159 *p* = 0.116			R^2^ = 0.049 R^2^ change = 0.017 *F* = 0.476 *p* = 0.702			R^2^ = 0.054 R^2^ change < 0.001 *F* = 0.529 *p* = 0.666			R^2^ = 0.040 R^2^ change = 0.014 *F* = 0.393 *p* = 0.759			R^2^ = 0.146 R^2^ change = 0.005 *F* = 1.597 *p* = 0.212		
Age	−0.373	0.123	**0.005**	−0.365	0.145	**0.018**	−0.158	0.164	0.346	−0.192	0.163	0.248	−0.211	0.228	0.361	−0.251	0.246	0.315
Gender	2.116	1.565	0.187	1.278	1.856	0.497	−0.734	2.098	0.729	1.052	2.075	0.616	1.727	2.905	0.557	−4.480	3.134	0.164
IIV Change	0.677	2.306	0.771	−2.665	2.878	0.363	−2.175	3.091	0.487	0.075	3.058	0.981	−2.743	4.280	0.527	1.908	4.618	0.683

*p-tau, phosphorylated tau; Aβ, amyloid beta; S.E., standard error; IIV, intraindividual cognitive variability; CBF, cerebral blood flow; IPL, inferior parietal lobe; ITG, inferior temporal gyrus; mOFC, medial orbitofrontal cortex; rMFG, rostral middle frontal gyrus. For gender, women are the reference group. Bold values are statistically significant (p < 0.05). IIV was calculated by using the intraindividual standard deviation across 6 z-scores.*

### Secondary Analyses Adjusting for Change in Mean Neuropsychological Performance

When secondary analyses also adjusted for change in mean neuropsychological performance, results remained qualitatively and statistically similar. That is, hierarchical linear regression models showed that after adjusting for age and gender, as well as for the main effects of IIV change and p-tau/Aβ status, there was a significant interaction between IIV change and p-tau/Aβ status for change in hippocampal CBF [β = −0.377, *p* = 0.020; Overall model: R^2^ = 0.256, F (6,46) = 2.643, *p* = 0.028] and a nearly-significant interaction for change in entorhinal CBF [β = −0.326, *p* = 0.051; Overall model: R^2^ = 0.188, F (6,46) = 1.773, *p* = 0.126]. The significant result for hippocampal CBF was not maintained when the FDR was applied.

## Discussion

In this sample of well-characterized, community-dwelling older adults free of dementia, we found that there was a significant interaction between IIV change and p-tau/Aβ status for entorhinal and hippocampal CBF change after adjusting for age and gender. Among p-tau/Aβ+ individuals, there was an association between an increase in IIV over 12 months and reduced CBF, whereas there were no significant associations between change in IIV and CBF among p-tau/Aβ– participants. The current findings indicate that an increase in IIV longitudinally is associated with a reduction in CBF in key brain regions implicated early in the progression of AD pathogenesis, suggesting that IIV is associated with subtle cerebrovascular changes among individuals who are presumed to be on the AD continuum given they are p-tau/Aβ+.

Several studies have shown that IIV is independent of mean cognitive performance (Kälin et al., 2014) and sensitive to future decline and neurodegeneration above and beyond mean cognitive performance (Bangen et al., 2019). Notably, when we adjusted for change in mean neuropsychological performance in secondary models, interactions between IIV change and AD biomarker status on CBF remained similar although the interaction was slightly attenuated and marginally significant for the entorhinal ROI.

Results from the few existing studies that have examined associations of IIV and AD biomarkers or neuropathology have been mixed. An autopsy study showed that higher IIV was significantly associated with more severe neurofibrillary tangle pathology and trended toward an association with more severe neuritic plaques (Malek-Ahmadi et al., 2017) whereas another study found that IIV was not associated with *in vivo* CSF-based markers of AD pathology cross-sectionally (Watermeyer et al., 2020). A unique aspect of the current study is the integration of longitudinal data, CSF AD biomarkers, and CBF. In the current study the p-tau/Aβ+ group showed a trend toward higher IIV at baseline and significantly higher IIV at follow-up. We also found that change in IIV over time was associated with reduced CBF in AD vulnerable regions in p-tau/Aβ+ individuals, suggesting that evolving pathology and increasing IIV may be occurring within this relatively short follow-up period of 1 year among individuals presumed to be on the AD continuum (i.e., p-tau/Aβ+). Notably, there are multiple methodological differences between the present study and that by Watermeyer et al. (2020) including our use of a longitudinal sample and focus on CBF as well as differences in calculation of the IIV index.

Interactions between IIV and biomarker positivity predicted longitudinal change in CBF in two *a priori* ROIs: the hippocampus and the entorhinal cortex. The current results dovetail with our previous studies showing that greater IIV at baseline predicted greater cerebral atrophy in the entorhinal cortex and hippocampus longitudinally (Bangen et al., 2019) as well as cross-sectional associations between higher IIV and reduced CBF in AD vulnerable regions (Holmqvist et al., under review). IIV has often been suggested to index cognitive control processes subserved by frontal regions (MacDonald et al., 2009), and previous studies have linked higher IIV to reduced frontal lobe integrity in populations including HIV (Jones et al., 2018) and healthy aging (Lovden et al., 2013). Although increasing IIV did not relate to perfusion in the two *a priori* frontal ROIs examined in the present study, our observation of effects in medial temporal regions is in line with the typical spread of neurofibrillary tangle pathology in AD, which appear early in the medial temporal lobe and later in parietal and frontal cortex (Braak and Braak, 1991). The current results together with our previous findings of higher IIV relating to AD biomarker status (Holmqvist et al., under review), medial temporal lobe neurodegeneration (Bangen et al., 2019), and reduced CBF (Holmqvist et al., under review) suggest that increased IIV may relate to both AD and cerebrovascular pathologies, and may be a sensitive although not necessarily specific marker of neurologic disease.

Some of our significant results did not survive FDR correction. Although, notably, the stratified analyses showed that IIV change accounted for approximately 20% and 35% of the variance in regional CBF change for the hippocampus and entorhinal cortex (R^2^ change = 0.198 and 0.349 for Block 2 of the models), respectively, within the p-tau/Aβ+ group. Notably, these effects may be interpreted as moderate and substantial for the hippocampus and entorhinal cortex, respectively (Cohen, 1988) and are specific to regions affected early within the AD pathophysiological process. Given the relatively small sample size of the p-tau/Aβ+ group (*n* = 21), these findings should be replicated in larger samples.

Existing studies have proposed different methods for calculating IIV indices. Consistent with several previous studies, IIV was examined as within-person variability across different neuropsychological measures (Gleason et al., 2018; Bangen et al., 2019; Watermeyer et al., 2020), whereas other studies have defined IIV as the inconsistency of trial performance across one task (Duchek et al., 2013; Lovden et al., 2013; Richards et al., 2019). These two measurements of IIV have been found to be correlated and associated with increasing age and cognitive decline (Hilborn et al., 2009). Neuropsychological tests that are commonly used in research and clinical settings were included in our dispersion score to enhance generalizability. Calculating IIV from one testing session is especially advantageous and applicable to clinical settings since it requires no alteration of standardized testing procedures (Holtzer et al., 2008). However, it should be noted that IIV indices may be influenced by relative differences in the sensitivity and score distributions of component tasks as well as possible floor or ceiling effects (Cherry et al., 2002; Jacobson et al., 2009). Our study was somewhat limited by the measures available within the ADNI study including the lack of sensitive visuospatial measures.

Strengths of the study include the well-characterized longitudinal sample of older adults, use of CSF based biomarkers to measure p-tau/Aβ, and use of a sensitive measure of IIV. IIV is low-cost, non-invasive, and can easily be implemented in research and clinical settings. ASL MRI has strengths given it is a non-invasive method of measuring CBF. ASL CBF has growing evidence as a useful marker of subtle cerebrovascular change and has been associated with poorer everyday functioning (Sanchez et al., 2020), faster rates of memory decline, neurodegeneration, and progression of small vessel disease (Bangen et al., 2021).

Limitations of the study include a homogeneous racial/ethnic distribution and a highly educated sample, so results may not be generalizable to groups with differing demographic characteristics. Replication in diverse cohorts will be critical. In addition, future studies should examine whether IIV predicts longitudinal changes in amyloid and tau accumulation in the brain to further validate IIV as a sensitive marker of decline. Future research is also needed to establish consistent operationalization of IIV and development of normative data to aid in interpretation of IIV values and establishment of thresholds to distinguish normal vs. abnormal values. We hypothesize that increasing IIV would be observed in preclinical and early prodromal stages of dementia, although once dementia becomes more severe and an individual performs poorly across all cognitive measures, IIV would decrease. Future longitudinal research is needed to confirm this and help determine typical vs. atypical trajectories of IIV over time.

In summary, our findings suggest that IIV is associated with subtle cerebrovascular brain changes among individuals who are p-tau/Aβ+. Future longitudinal studies with longer follow up and larger samples may determine whether IIV may be a useful marker of dementia risk above and beyond mean-level neuropsychological performance and may be beneficial in identifying participants for clinical trials that target vascular risk or AD biomarkers.

## Data Availability Statement

Publicly available datasets were analyzed in this study. This data can be found here: http://adni.loni.usc.edu/.

## Ethics Statement

The studies involving human participants were reviewed and approved by the institutional review board at each participating site. The patients/participants provided their written informed consent to participate in this study.

## Author Contributions

SH and KB designed the study, performed the statistical analyses, interpreted the data, and drafted the manuscript. KT organized the database, assisted with statistical analyses, interpreted the data, and performed critical review of the manuscript. EB, EE, AC, LE, and MB performed the critical review of the manuscript. All authors contributed to the article and approved the submitted version.

## Author Disclaimer

The content is solely the responsibility of the authors and does not necessarily represent the official views of the National Institutes of Health.

## Conflict of Interest

The authors declare that the research was conducted in the absence of any commercial or financial relationships that could be construed as a potential conflict of interest.

## Publisher’s Note

All claims expressed in this article are solely those of the authors and do not necessarily represent those of their affiliated organizations, or those of the publisher, the editors and the reviewers. Any product that may be evaluated in this article, or claim that may be made by its manufacturer, is not guaranteed or endorsed by the publisher.

## Footnotes

1
adni.loni.usc.edu

2
surfer.nmr.mgh.harvard.edu

3
http://www.fil.ion.ucl.ac.uk/spm/
