# Supplementary figures and images for: Longitudinal Intraindividual Cognitive Variability Is Associated With Reduction in Regional Cerebral Blood Flow Among Alzheimer’s Disease Biomarker-Positive Older Adults
Source: Front Aging Neurosci. 2022 Jul 6;14:859873. doi: 10.3389/fnagi.2022.859873 (PMC9300445; doi:10.3389/fnagi.2022.859873)

## Supplemental Materials

Figure S1. Study sample flow chart

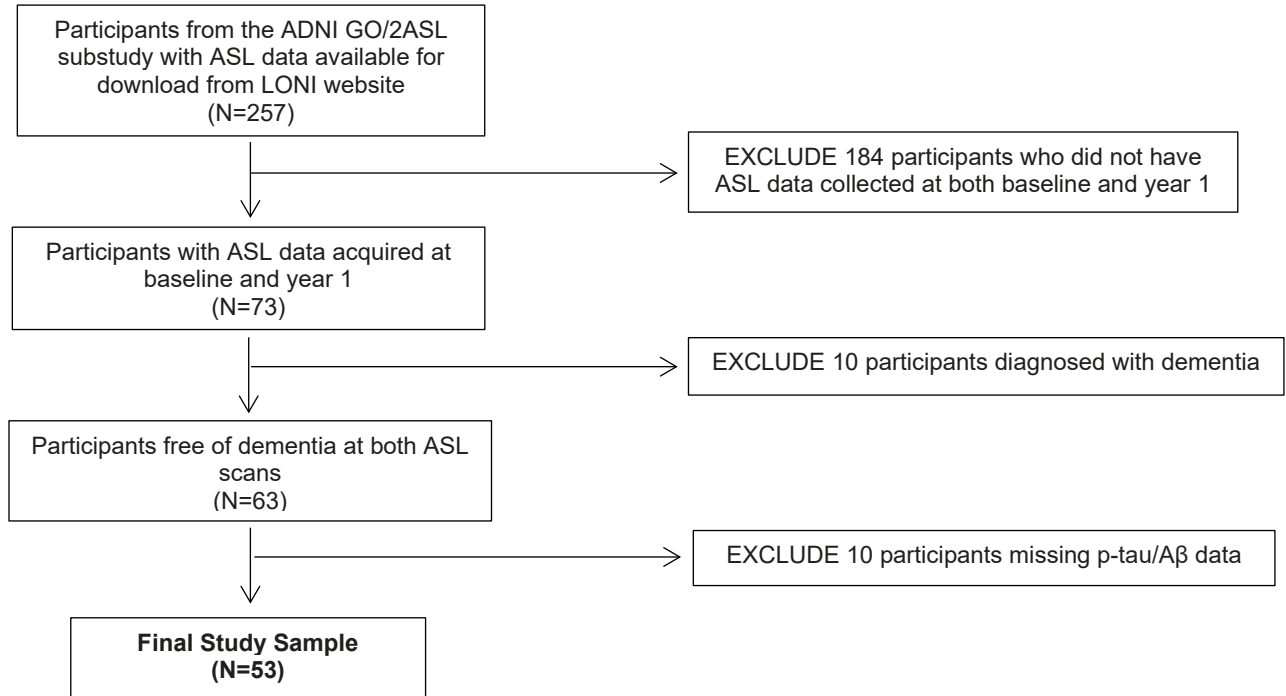

Supplement: Supplementary file 1 [file Image_1.pdf]
